# Correlation of noninvasive imaging of tumour-infiltrating lymphocytes with survival and BCG immunotherapy response in patients with bladder cancer: a multicentre cohort study

**DOI:** 10.1097/JS9.0000000000001999

**Published:** 2024-08-05

**Authors:** Ke Chen, Xiaoyang Li, Libo Liu, Bo Wang, Weiming Liang, Junyu Chen, Mingchao Gao, Xiaodong Huang, Bohao Liu, Xi Sun, Tenghao Yang, Xiao Zhao, Wang He, Yun Luo, Jian Huang, Tianxin Lin, Wenlong Zhong

**Affiliations:** a Department of Urology, Sun Yat-sen Memorial Hospital, Sun Yat-sen (Zhongshan) University; b Department of Urology, The Third Affiliated Hospital of Sun Yat-sen University, Sun Yat-sen (Zhongshan) University, Guangzhou; c Department of Urology, Shenzhen People's Hospital (The Second Clinical Medical College, Jinan University; The First Affiliated Hospital, Southern University of Science and Technology), Shenzhen, China; d Guangdong Provincial Key Laboratory of Malignant Tumour Epigenetics and Gene Regulation, Guangdong-Hong Kong Joint Laboratory for RNA Medicine, Medical Research Center, Sun Yat-sen Memorial Hospital, Sun Yat-sen (Zhongshan) University, Guangzhou; e Department of Urology, The First Affiliated Hospital of Guangxi University of Science and Technology, Guangxi University of Science and Technology, Liuzhou, People’s Republic of China

**Keywords:** bacillus Calmette-Guérin, bladder cancer, computed tomography, immunotherapy, radiomics, tumour-infiltrating lymphocyte

## Abstract

**Background:**

Tumour-infiltrating lymphocytes (TILs) are strongly correlated with the prognosis and immunotherapy response in bladder cancer. The TIL status is typically assessed through microscopy as part of tissue pathology. Here, the authors developed Rad-TIL model, a novel radiomics model, to predict TIL status in patients with bladder cancer.

**Material and methods:**

The authors enrolled 1089 patients with bladder cancer and developed the Rad-TIL model by using a machine-learning method based on computed tomography (CT) images. The authors applied a radiogenomics cohort to reveal the key pathways underlying the Rad-TIL model. Finally, the authors used an independent treatment cohort to evaluate the predictive efficacy of the Rad-TIL model for Bacillus Calmette-Guérin (BCG) immunotherapy.

**Results:**

The authors developed the Rad-TIL model by integrating tumoral and peritumoral features on CT images and obtained areas under the receiver operating characteristic curves of 0.844 and 0.816 in the internal and external validation cohorts, respectively. Patients were stratified into two groups based on the predicted radiomics score of TILs (RS_TIL_). RS_TIL_ exhibited prognostic significance for both overall and cancer-specific survival in each cohort (hazard ratios: 2.27–3.15, all *P*<0.05). Radiogenomics analysis revealed a significant association of RS_TIL_ with immunoregulatory pathways and immune checkpoint molecules (all *P*<0.05). Notably, BCG immunotherapy response rates were significantly higher in high-RS_TIL_ patients than in low-RS_TIL_ patients (*P*=0.007).

**Conclusion:**

The Rad-TIL model, a noninvasive method for assessing TIL status, can predict clinical outcomes and BCG immunotherapy response in patients with bladder cancer.

## Introduction

Highlights

- We employed a CT radiomics model using a machine-learning method to distinguish the lymphocyte infiltration status in bladder cancer.
- The Rad-TIL model, a noninvasive method for assessing TIL status, can predict clinical outcomes and BCG immunotherapy response in patients with bladder cancer.
- The model can assist clinicians in future assessments of the tumour immune environment and individual treatment decision-making related to bladder cancer.

Bladder cancer is currently the ninth most prevalent and the 13th most fatal cancer globally^1,2^. Among various cancer types, bladder cancer has a large overall mutation burden and a high immunogenic potential; this highlights the significant role of immunotherapy in bladder cancer management^3,4^. Bladder cancer is categorised as nonmuscle-invasive bladder cancer (NMIBC) and muscle-invasive bladder cancer (MIBC)^5^. Approximately 75% of patients present with NMIBC^5^. Bacillus Calmette-Guérin (BCG) immunotherapy has emerged as a golden standard for the management of high-risk NMIBC cases^6^. Partial patients with bladder cancer can derive benefits from BCG immunotherapy; however, the substantial heterogeneity of bladder cancer and the absence of robust predictive methods have impeded the effective implementation of personalised treatment strategies^7,8^. Therefore, before treatment initiation, ascertaining which patients are likely to benefit from BCG immunotherapy is imperative in order to avoid administering ineffective treatments.

Tumour-infiltrating lymphocytes (TILs) provide insight into the host immune responses against cancer cells^9,10^, making it possible to predict the immunotherapy response status of patients. TILs and interferon γ signalling pathway activation are crucial prerequisites for tumour immunotherapy effectiveness^11,12^. TILs—generally assessed using haematoxylin and eosin (H&E) staining—are valuable prognostic indicators in various solid tumours^13^. We previously confirmed that stromal TILs serve as reliable prognostic markers in patients with bladder cancer^14^. Nevertheless, evaluating TILs necessitates the acquisition of histopathological tissue via surgical or other invasive procedures. Moreover, the assessment of TILs through histopathology is limited by spatial heterogeneity and temporal evolution, which renders result interpretation complex. Consequently, there is a pressing clinical demand for a noninvasive method to evaluate TILs and thus assist clinical physicians in making informed treatment decisions.

Radiomics can address these limitations by noninvasively capturing comprehensive information regarding the entire tumour burden^15^. Assessment through radiomic imaging may be feasible and representative of the entire tumour immune microenvironment (TIME), making it a potential alternative to tissue-based assessment of TILs through biopsy or surgery^15–17^. The strong correlation between the radiomic features and the TIME indicates that radiomic evaluation of the TIME has a wide range of applications in guiding clinical decision-making. However, no study thus far has focused on the predictive role of radiomics in bladder cancer TIME, and its association with the effects of immunotherapy in patients with bladder cancer remains unexplored.

In this study, we developed the Rad-TIL model, a radiomics model that predicts TIL status by combining computed tomography (CT) imaging features from both tumoral and peritumoral regions. We evaluated the model’s association with prognosis and BCG immunotherapy response in patients with bladder cancer. In addition, we integrated transcriptomics data to reveal the biological relevance inherent in the Rad-TIL model.

## Material and methods

**Figure 1:**
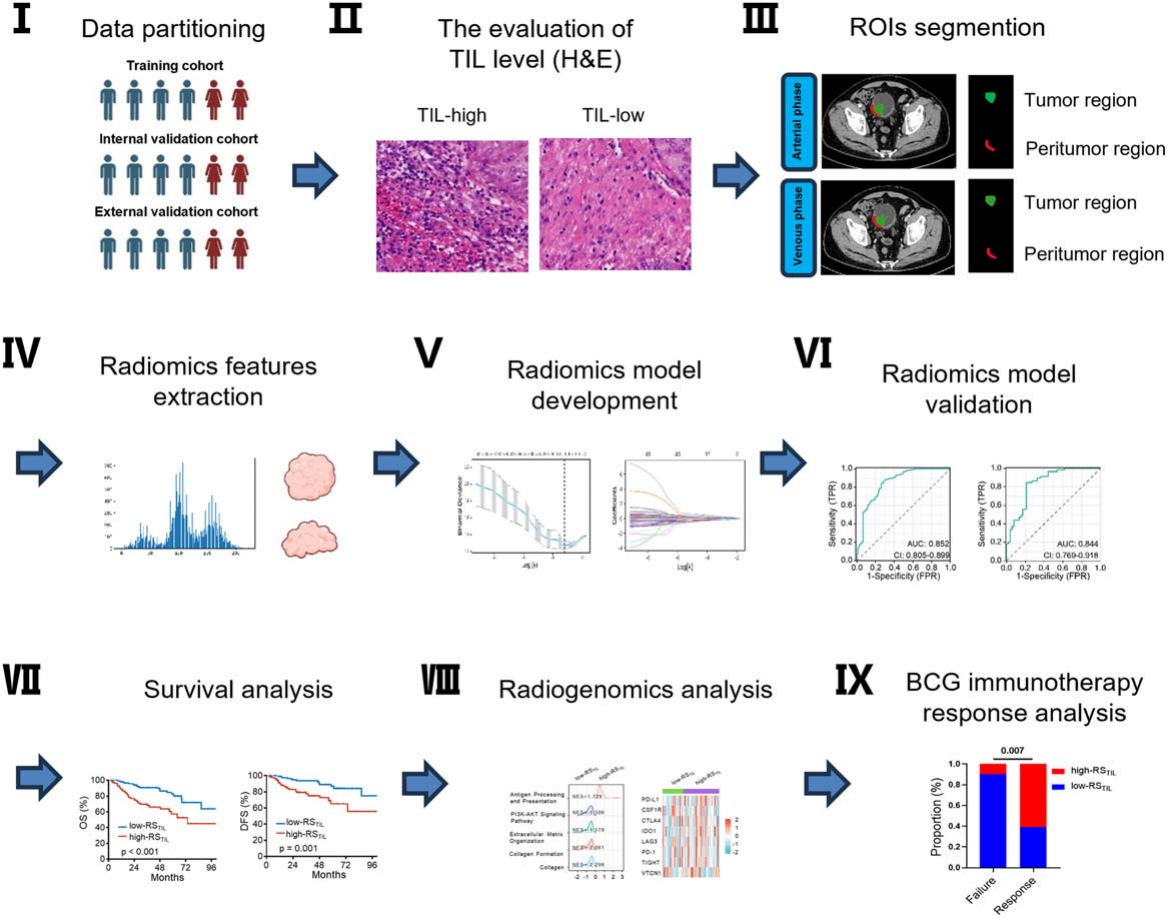
Study flowchart.

Figure 1 presents the flow of our study process.

## Patients and tissue specimens

We only included patients with (a) histologically confirmed primary bladder cancer, (b) treated with transurethral resection of bladder tumour or radical cystectomy, (c) having preoperative pelvic CT-enhanced images, and (d) having complete clinicopathological data. Exclusion criteria included (a) distant metastases at diagnosis, (b) history of other synchronous malignancies or surgical history of malignancies, (c) combined upper urinary tract urothelial carcinoma, and (d) no primary tumour detected on CT.

**Figure 2:**
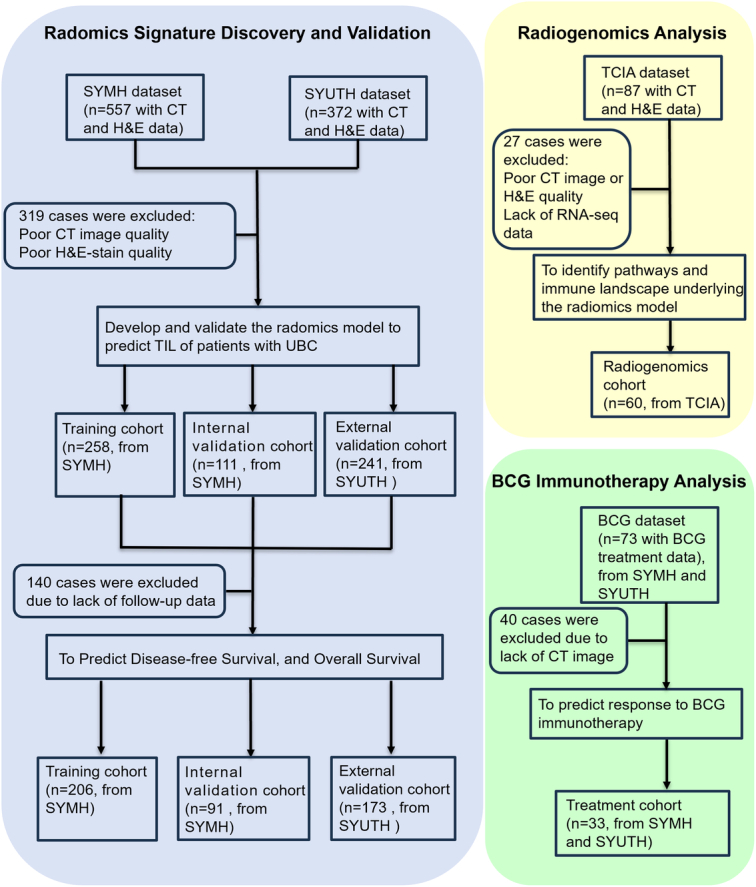
Patient enrolment process. The training, internal validation, and external validation cohorts, recruited from SYMH and SYUTH, were used for developing and validating the radiomics model. This model aimed to predict TIL status and assess its correlation with cancer-specific survival and overall survival. The treatment cohort with BCG immunotherapy data was used to assess the correlation of the radiomics model with the BCG immunotherapy response rates. The radiogenomic cohort, recruited from The Cancer Imaging Archive (TCIA) dataset, was employed to identify biological pathways and the immune landscape underlying the radiomics model. BCG, Bacillus Calmette-Guérin; SYMH, Sun Yat-sen Memorial Hospital; SYUTH, Third Affiliated Hospital of Sun Yat-sen University; TIL, tumour-infiltrating lymphocyte.

The patient enrolment process is shown in Figure 2. First, to assess the efficiency of the Rad-TIL model in predicting TILs and survival, we retrospectively reviewed CT imaging and H&E staining data of 929 patients from two medical centres: Sun Yat-sen Memorial Hospital (SYMH; n = 557) and the Third Affiliated Hospital of Sun Yat-sen University (SYUTH; n = 372). These data were collected from October 2010 to September 2020. After screening, we divided 369 patients from SYMH randomly into a training cohort (*n*=258) and an internal validation cohort (*n*=111) at a 7:3 ratio. We included 241 patients who met the inclusion criteria but did not meet the exclusion criteria from SYUTH as an external validation cohort. After the exclusion of patients without follow-up data, the remaining patients, including those from the training (*n*=206), internal validation (*n*=91), and external validation (*n*=173) cohorts, were used for survival analysis. Second, we collected a radiogenomics cohort (*n*=60) with paired CT imaging and RNA sequencing (RNA-seq) data from The Cancer Imaging Archive (TCIA) database (*n*=87) to identify the biological pathways and immune landscape underlying the Rad-TIL model. Third, to assess the predictive value of the Rad-TIL model for BCG immunotherapy response, we extracted an independent treatment cohort (*n*=33) with CT imaging and BCG immunotherapy data from two medical centres (*n*=52 from SYMH and 21 from SYUTH) between January 2016 and June 2020. This study was approved by the institutional ethical committees of both SYMH (approval number: SYSKY-2023-467-01) and SYUTH (approval number: II2023-303-02). This trial was registered on the ClinicalTrials network (http://www.clinicaltrial.gov) under the identifier NCT06381895. The work is reported in line with the strengthening the reporting of cohort, cross-sectional, and case–control studies in Surgery (STROCSS) (Supplemental Digital Content 1, http://links.lww.com/JS9/D244) criteria^18^. The requirement for informed consent was waived due to this study’s retrospective and anonymous nature.

### TILs evaluation

TILs were evaluated using paraffin-embedded tumour tissue samples stained with H&E according to established protocols^14,19^. Slides were observed under a Leica scanner (Wetzlar, Germany) at 400× magnification, and images were visualised using ImageScope (version 12.4.6; Aperio, Vista, CA, USA). Two experienced pathologists independently evaluated TIL levels by using the assessment criteria of the Immuno-Oncology International TIL Working Group (https://www.tilsinbreastcancer.org/)^20^. Both pathologists were blinded to the clinical data and reached consensus in cases of discordant results. The assessment slides were selected to represent the most invasive part, consistent with routine pathology for determining the tumour stage.

TILs were comprehensively assessed across the entire slide (nonhot spot), with all mononuclear cells in the tumour stromal regions considered TILs. The TIL level was estimated as a percentage of the total stromal area within the tumour, with analysis conducted in 10 percentage point increments (10%, 20%, 30%, etc.). TILs in areas of necrosis, outside the tumour border, or within carcinoma in situ were excluded. Subsequently, samples were categorised as having low (≤20%) or high (>20%) infiltration based on the TIL percentages in line with previous studies^21,22^. Representative images of stromal TILs in high-density and low-density regions are shown in online Supplemental Figure S1 (Supplemental Digital Content 2, http://links.lww.com/JS9/D245).

### CT image acquisition, segmentation, and radiomics features extraction

Before the surgical intervention, all patients underwent a pelvic contrast-enhanced CT scan on a 64-row spiral CT scanner (Somatom Sensation 64; Siemens Medical Systems, Erlangen, Germany). This scan included a nonenhanced CT scan, followed by a dynamic contrast-enhanced CT scan with 80–100 ml of intravenous nonionic contrast agent. The images for the arterial and venous phases were acquired within 25 and 60 s, respectively, with a slice thickness of 1.0 mm for the reconstructed images. Subsequently, the arterial-phase and venous-phase CT images were obtained in the DICOM format from each institution’s picture archiving and communication system and subsequently converted to the NIFFTI format for further processing.

An experienced radiologist used ITK-SNAP (version 3.8.0) to delineate the regions of interest (ROIs) corresponding to the primary tumour sites on the CT images. For each case, four ROIs were defined, with the tumoral and peritumoral regions originating from both arterial and venous CT images. The tumoral region was manually segmented on the largest cross-section of the tumour, with complete exclusion of necrotic areas, calcifications, and tumour margins. The peritumoral region was subsequently created by automatically expanding the tumour boundary 2 mm outward and contracting it 1 mm inward. Portions protruding toward the bladder cavity were excluded. Consequently, for each patient, four ROIs were obtained, which were identified as the tumoral and peritumoral regions in the arterial-phase (A_TR and A_PR, respectively), as well as the tumoral and the peritumoral regions in the venous-phase (V_TR and V_PR, respectively).

Radiomics features within the ROIs were extracted using Python. To mitigate the impact of inconsistent CT spatial resolution, all images were resampled to 1.0 mm pixels in all three anatomical directions. Subsequently, the images were preprocessed; it included both high-pass and low-pass filtering by using wavelet filters and coarse or complex texture enhancement by employing Laplace Gaussian filters with various σ parameters. A comprehensive set of seven types of image features were extracted: (1) first-order, (2) shape, (3) grey-status cooccurrence matrix, (4) grey-status dependence matrix, (5) grey-status run-length matrix, (6) grey-status size-zone matrix, and (7) neighbourhood grey-zone difference matrix features. For feature selection and model training, the data were standardised using the StandardScaler algorithm from the scikit-learn package.

### Rad-TIL model development

The Mann–Whitney *U* test and maximum relevance minimum redundancy (mRMR) algorithm were initially employed to eliminate irrelevant, redundant features. Subsequently, the least absolute shrinkage and selection operator (LASSO) analysis was employed to select the optimal subset of TIL-related features with nonzero coefficients. Based on four ROI categories (A_TR, A_PR, V_TR, and V_PR), six subgroups of TIL-related features were selected for the development of six corresponding TIL prediction models. They encompassed four fundamental models (A_TR, A_PR, V_TR, and V_PR models) and two composite models (A_TR+PR and V_TR+PR models). To construct the six TIL prediction models, we used support vector machine (SVM) classifiers by leveraging different subsets of TIL-related features selected through LASSO analysis. Moreover, we used the synthetic minority oversampling technique to address data imbalances between high- and low-TIL groups during model fitting.

To assess the predictive efficacy of the radiomics model in determining TIL status, we employed receiver operating characteristic (ROC) analysis and concurrently calculated the area under the curve (AUC). To determine the best-performing TIL prediction model, we compared the AUCs of the six TIL prediction models by using the DeLong test in both the training and internal validation cohorts. Among the six models, we identified the highest-performing model and designated it as the Rad-TIL model. In the Rad-TIL model, the patients were stratified into two groups on the basis of the predicted the radiomics score of TILs (RS_TIL_).

### Prognosis and BCG immunochemotherapy response analyses

To evaluate the prognostic significance of the Rad-TIL model, we examined overall survival (OS) and cancer-specific survival (CSS) across distinct RS_TIL_ groups in the training, internal validation, and external validation cohorts by using Kaplan–Meier survival curves. OS was defined as the time from surgery until the occurrence of death, whereas CSS was defined as the time from surgery until the occurrence of death due to bladder cancer.

To enhance our understanding of the predictive potential of the Rad-TIL model for BCG immunotherapy, we examined the classification utility of RS_TIL_ within the treatment cohort. The minimum follow-up duration in all patients who underwent BCG immunotherapy was 2 years. Objective response rates were evaluated during the follow-up period and categorised as either ‘failure’ (indicating tumour recurrence) or ‘response’ (indicating no tumour recurrence). Tumour recurrence was defined as confirmation of the presence of tumours in the cystoscopy biopsy results or a positive urinary cytology examination during the follow-up process.

### Biological pathway and immune landscape analyses

Potential biological pathways and immune landscape associated with the Rad-TIL model were discerned through an analysis of the radiogenomics cohort. The DESeq2 package was employed to detect differentially expressed genes (DEGs) in the high-RS_TIL_ and low-RS_TIL_ subgroups. The DEG inclusion criteria were changes in gene expression with |log_2_(fold change)| ≥ 1. Gene set enrichment analysis (GSEA) was performed to assess the enrichment of gene expression in various biological pathways among different RS_TIL_ groups, with the criterion of *P*<0.05 and false discovery rate <0.25. Visualisation was facilitated using the cluster profile and the ggplot2 package.

### Immunohistochemistry analyses and evaluation

Formalin-fixed, paraffin-embedded tissue samples (*n*=173) from the SYMH cohort were subjected to immunohistochemical (IHC) analysis by using previously established protocols^19,23^. In brief, 5 μm-thick tissue sections were dewaxed and rehydrated, followed by antigen retrieval, endogenous peroxidase inactivation, and nonspecific binding blockade. The sections were incubated with antibodies against CD8 (1:500; Thermo Fisher Scientific) and programmed death ligand 1 (PD-L1; 1:200; Cell Signalling Technology) at 4°C overnight. The sections were subsequently incubated with the corresponding secondary antibodies (Vector) and stained with peroxidase and 3,3′-diaminobenzidine tetrahydrochloride in the EnVision Detection System (DAKO). Finally, the slides were counterstained with haematoxylin, visualised in 400× high-power fields under an ECLIPSE Ni‑E/Ni‑U microscope (Nikon), and assessed manually.

PD-L1 expression on tumour cells (TCs) was assessed and regarded as negative or positive. A staining pattern of <5% in the IHC analysis was considered negative, whereas that of ≥5% was considered positive^23^. CD8^+^ TIL densities were assessed at five representative high-power fields for each specimen (magnification, 400×; 0.07 mm^2^/field) and counted manually. Two independent pathologists, blind to clinicopathological data, performed the assessment.

### Statistical analysis

Statistical analysis was conducted using Python (version 3.0), R (version 4.2.1), GraphPad Prism (version 9.0), and SPSS (version 23.0; IBM). Continuous variables were compared using the Mann–Whitney *U* test, whereas categorical variables were compared using the Pearson *χ* ^2^ or Fisher exact test, as appropriate. Cumulative survival time was calculated to assess prognostic significance by employing the Kaplan–Meier method along with the log-rank test. Propensity score matching was employed to reduce the impact of potential confounders by performing 1:2 optimal pair matching using age, sex, tumour multifocality, tumour size, and pathologic T stage. Data are presented as means±standard errors of means (SEM), and statistical significance was set at *P*<0.05.

## Results

### Rad-TIL model development and validation

We enrolled 258 and 111 patients from SYMH in our training and internal validation cohorts, respectively. Moreover, our external cohort comprised 241 patients from SYUTH. Clinicopathological characteristics of patients in the training, internal validation, and external validation cohorts were presented in the online Supplemental Table S1 (Supplemental Digital Content 2, http://links.lww.com/JS9/D245). No significant differences were observed among the cohorts, except for higher unifocal tumour prevalence in the external validation cohort (77.6%) compared with that in the training and internal validation cohorts (49.6 and 47.7%, respectively; *P*<0.001).

**Figure 3:**
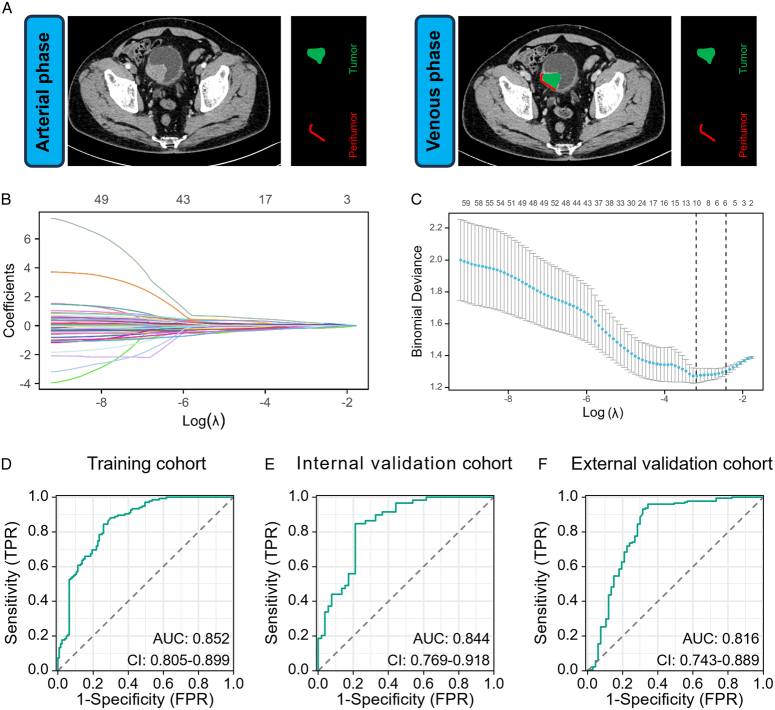
Construction and validation of a novel Rad-TIL model in patients with bladder cancer. (A) Segmentation of ROIs in CT images of the arterial and venous phases. For each case, four ROIs were obtained: the tumoral and peritumoral regions in the arterial-phase (A_TR and A_PR, respectively), as well as the tumoral and the peritumoral regions in the venous-phase (V_TR and V_PR, respectively). The tumoral region was manually segmented on the largest cross-section of the tumour, whereas the peritumoral region was automatically expanded 2 mm outward from the tumour boundary and contracted 1mm inward (while eliminating the part protruding toward the bladder cavity). (B) LASSO coefficient profiles of the TIL-related features. (C) Selection of the most optimal TIL-related features using LASSO logistic regression. (D-F) AUCs demonstrating Rad-TIL model performance in the training, internal validation, and external validation cohorts. AUC, area under the curve; CT, computed tomography; LASSO, least absolute shrinkage and selection operator; ROIs, regions of interest; ROC, receiver operating characteristic; TIL, tumour-infiltrating lymphocyte.

After CT image acquisition and ROI segmentation, we obtained four ROIs: A_TR, A_PR, V_TR, and V_PR (Fig. 3A). In total, 567 radiomics features were extracted from the ROIs corresponding to A_TR, A_PR, V_TR, and V_PR. By using an SVM classifier, we developed six TIL prediction models, comprising four individual models (A_TR model, A_PR model, V_TR model, V_PR model) and two composite models (A_TR+PR model, and V_TR+PR model). Online supplemental Table S2 (Supplemental Digital Content 2, http://links.lww.com/JS9/D245) presents the TIL-related radiomics features and their corresponding coefficients used in the different models. Table 1 displays the performance metrics (sensitivity, specificity, positive predictive value, and negative predictive value), as well as the AUCs, for predicting TIL status in both the training and internal validation cohorts for these models. Compared with those of the remaining five models, the AUCs of the V_TR+PR model were significantly higher in both the training and internal validation cohorts (Table 1).

**Table 1:** The performance of different models for evaluating TIL status.

	Training cohort						Internal validation cohort					
Models	Sensitivity	Specificity	PPV	NPV	AUC	*P*	Sensitivity	Specificity	PPV	NPV	AUC	*P*
A_TR model	91.1%	60.9%	71.9%	86.2%	0.826	0.279	93.3%	36.5%	62.5%	82.6%	0.654	<0.001
A_PR model	68.8%	65.8%	68.8%	65.8%	0.715	<0.001	64.4%	67.3%	69.0%	62.5%	0.646	<0.001
V_TR model	78.5%	68.2%	73.1%	74.3%	0.771	<0.001	79.6%	71.1%	75.8%	75.5%	0.782	0.019
V_PR model	82.9%	89.4%	89.6%	82.7%	0.912	0.021	89.8%	42.2%	64.6%	79.3%	0.658	<0.001
A_TR+PR model	70.3%	70.7%	72.5%	68.5%	0.776	0.002	77.9%	61.5%	69.6%	71.1%	0.678	<0.001
V_TR+PR model	84.4%	73.9%	78.0%	81.2%	0.852	Ref	84.7%	78.8%	81.9%	82.0%	0.844	Ref

A, arterial phases; AUC, area under curve; NPV, negative predictive value; PPV, positive predictive value; PR, peritumoral region; TIL, tumour-infiltrating lymphocyte; TR, tumoral region; V, venous phases.

Significant *P*-values are shown in bold.

The V_TR+PR model, demonstrating superior TIL status prediction performance, was chosen and named the Rad-TIL model. In total, 10 features with the minimum lambda values were selected for the development of the Rad-TIL model through LASSO analysis (Fig. 3B and C). These features comprised seven from V_TR and three from V_PR (online Supplemental Table S2, Supplemental Digital Content 2, http://links.lww.com/JS9/D245). Notably, the Rad-TIL model exhibited excellent predictive performance—with AUCs of 0.852 (95% CI: 0.805–0.899; Fig. 3D), 0.844 (95% CI: 0.769–0.918; Fig. 3E), and 0.816 (95% CI: 0.743–0.889; Fig. 3F) in the training, internal validation, and external validation cohorts, respectively.

### Correlation of Rad-TIL model with clinicopathological parameters and survival

We examined the relationships between the Rad-TIL model of the clinicopathological parameters and survival in the training, internal validation, and external validation cohorts. The patients were stratified into high-RS_TIL_ and low-RS_TIL_ groups based on their RS_TIL_ status. In the training cohort, high-RS_TIL_ was positively associated with aggressive clinicopathological features, such as larger tumour size, higher T stage, higher N stage, and higher histological grade (all *P*<0.001; online Supplemental Table S3, Supplemental Digital Content 2, http://links.lww.com/JS9/D245). In the internal validation cohort, a positive correlation was observed between tumour size and RS_TIL_ (*P*=0.002), whereas RS_TIL_ exhibited a positive correlation with tumour size (*P*<0.001), T stage (*P*<0.001), N stage (*P*=0.041), and histological grade (*P*<0.001) in the external validation cohort.

**Figure 4:**
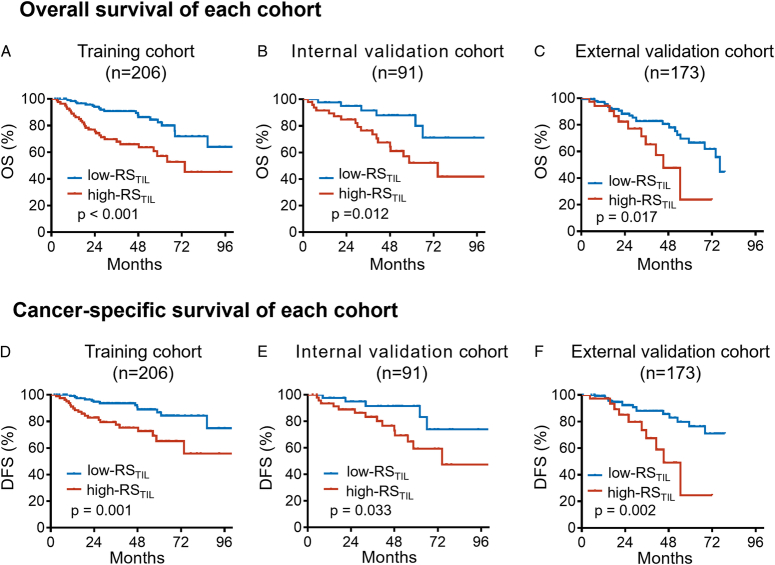
Kaplan–Meier analysis of survival outcomes in different RS_TIL_ groups in patients with bladder cancer. Kaplan–Meier curves depicting (A-C) OS and (D-F) DFS in different RS_TIL_ groups in the training, internal validation, and external validation cohorts. DFS, disease-free survival; OS, overall survival; RS_TIL_, radiomics score of tumour-infiltrating lymphocyte.

In the training, internal validation, and external validation cohorts, the median (IQR) follow-up durations were 46.7 [interquartile range (IQR) 25.6–61.6)], 49.9 (IQR 32.5–62.1), and 24.8 (IQR 14.8–44.3), respectively. Moreover, high-RS_TIL_ was positively associated with inferior OS and CSS in all three cohorts (all *P*<0.05; Fig. 4A-F). The 5-year OS rates were significantly higher in the low-RS_TIL_ group—reaching 86.2, 87.9, and 77.9% in the training, internal validation, and external validation cohorts, respectively. In contrast, they were significantly lower in the high-RS_TIL_ groups—reaching 63.6, 61.0, and 47.6% in the training, internal validation, and external validation cohorts, respectively. Similar findings were also observed for the 5-year CSS rates for each cohort; the 5-year CSS rates in the training, internal validation, and external validation cohorts were, respectively, 88.9, 91.4, and 82.8% among the low-RS_TIL_ patients and 72.6, 67.3, and 49.1% among the high-RS_TIL_ patients.

### Biological pathways and immune landscape underlying Rad-TIL model

The clinicopathological characteristics of the patients in the radiogenomics cohort are listed in the online Supplemental Table S4 (Supplemental Digital Content 2, http://links.lww.com/JS9/D245). Our initial investigation focused on revealing the potential biological pathways and immune landscape associated with TILs by using H&E staining classification in the radiogenomics cohort. The GSEA results indicated the enrichment of numerous immune regulation pathways in different TIL levels in H&E staining (online Supplemental Figure S2A, Supplemental Digital Content 2, http://links.lww.com/JS9/D245). Patients with high TIL levels in H&E staining also exhibited elevated immune checkpoint molecule expression and CD8^+^ cell infiltration (online Supplemental Figure S2B and S2C, Supplemental Digital Content 2, http://links.lww.com/JS9/D245).

**Figure 5:**
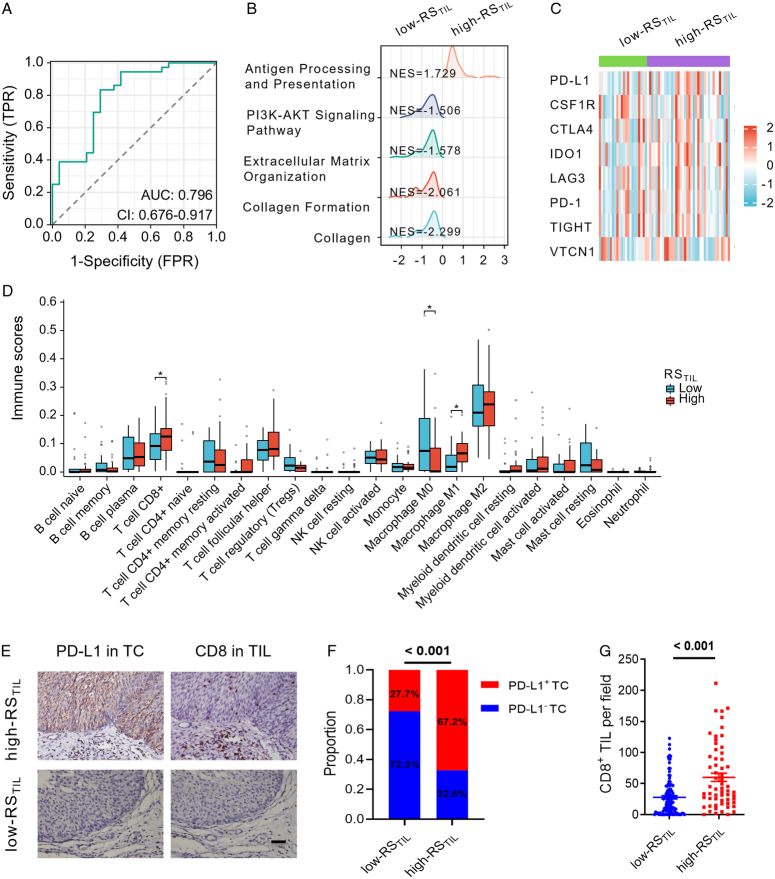
Identification of biological pathways and immune landscape underlying the Rad-TIL model. (A) AUCs demonstrating Rad-TIL model performance in predicting TILs in the radiogenomic cohort. (B) Ridgeline plots displaying GSEA results for representative immune-related pathways enriched in different RS_TIL_ groups in the radiogenomic cohort. (C) Heatmap demonstrating immune checkpoint molecule expression with different RS_TIL_ groups in the radiogenomic cohort. (D) Analysis of immune cell infiltration differences in different RS_TIL_ groups using the CIBERSORT algorithm in the radiogenomic cohort. (E) Representative images of IHC staining of PD-L1 in TCs (brown) and CD8^+^ TIL infiltration (brown) on consecutive sections of bladder cancer tissue samples. Scale bars: 50 μm. (F) Association between RS_TIL_ and PD-L1^+^ TC proportions in bladder cancer tissues. (G) Association between RS_TIL_ and CD8^+^ TIL infiltration levels in bladder cancer tissues. AUC, area under the ROC curve; ROC, receiver operating characteristic; GSEA, gene set enrichment analysis; TIL, tumour-infiltrating lymphocyte; RS_TIL_, radiomics score of tumour-infiltrating lymphocyte; PD-L1, programmed death ligand 1.

We subsequently conducted a comprehensive analysis of the biological pathways and immune landscape associated with the Rad-TIL model in the radiogenomics cohort, consisting of 60 patients with both paired CT image and RNA-seq data. The AUC for identifying the TIL status in the radiogenomics cohort was 0.796 (95% CI: 0.676–0.917; Fig. 5A). Significant GSEA terms were visualised using ridgeline plots (Fig. 5B). The GSEA results demonstrated that the ‘Antigen Processing and Presentation’ pathway was significantly enriched in the high-RS_TIL_ group, whereas the immune suppression pathways including the ‘PI3K-AKT Signalling’, ‘Extracellular Matrix Organisation’, ‘Collagen Formation’, and ‘Collagen’ pathways were significantly enriched in the low-RS_TIL_ group. These GSEA findings suggested that patients in the high-RS_TIL_ group tended to display a more immunologically activated state than the low-RS_TIL_ group. Furthermore, several immune checkpoint molecules, including PD-L1, cytotoxic T-lymphocyte-associated protein 4 (CTLA-4), and indoleamine-pyrrole 2,3-dioxygenase 1 (IDO-1), displayed strong expression in the high-RS_TIL_ group (Fig. 5C). Discrepancies in the immune cell composition within the TIME were observed between high-RS_TIL_ and low-RS_TIL_ patients. The CIBERSORT results indicated significantly higher CD8^+^ T-cell and macrophage M1 scores but significantly lower macrophage M0 scores were noted in high-RS_TIL_ patients (Fig. 5D).

We subsequently explored the associations between the Rad-TIL model and the PD-L1/CD8 axis through IHC staining in a cohort comprising 173 individuals from SYMH. Representative images obtained from IHC staining of consecutive sections demonstrated that PD-L1^+^ tumour cells (TCs) and CD8^+^ TILs were predominantly present in high-RS_TIL_ patients; in contrast, PD-L1^+^ TCs and CD8^+^ TILs were seldom detected in low-RS_TIL_ patients (Fig. 5E). The proportion of patients exhibiting PD-L1^+^ TCs was significantly higher in the high-RS_TIL_ group than in the low-RS_TIL_ group (67.2% vs. 27.7%; *P*<0.001; Fig. 5F). Similarly, CD8^+^ TILs were more frequent in the high-RS_TIL_ group than in the low-RS_TIL_ group (59.8±0.8 vs. 28.7±0.2 cells/field; *P*<0.001; Fig. 5G).

### BCG immunotherapy response predictive value of Rad-TIL model

BCG immunotherapy has emerged as a golden standard for the management of high-risk NMIBC cases. Recurrence-free survival (RFS) was compared between patients who received intravesical BCG immunotherapy and those who received intravesical infusion chemotherapy. Following a 1:2 propensity score matching for clinicopathological characteristics (Table S6), our analysis revealed that intravesical BCG immunotherapy was associated with superior RFS (online Supplemental Figure S3A, Supplemental Digital Content 2, http://links.lww.com/JS9/D245). Interestingly, within the BCG immunotherapy group, high TIL was positively associated with better RFS (online Supplemental Figure S3B, Supplemental Digital Content 2, http://links.lww.com/JS9/D245), while no significant disparities were observed in the infusion chemotherapy group (online Supplemental Figure S3C, Supplemental Digital Content 2, http://links.lww.com/JS9/D245).

**Figure 6:**
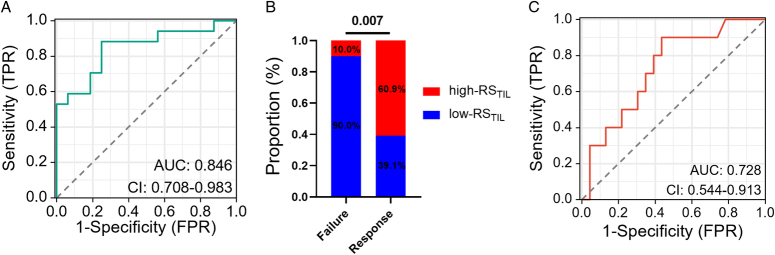
Rad-TIL model performance in distinguishing BCG immunotherapy response. (A) AUCs demonstrating Rad-TIL model performance in predicting TIL status. (B) Association between RS_TIL_ status and BCG immunotherapy response. (C) AUCs demonstrating Rad-TIL model performance in distinguishing BCG immunotherapy responses. AUC, area under the ROC curve; BCG, Bacillus Calmette-Guérin; ROC, receiver operating characteristic; RS_TIL_, radiomics score of tumour-infiltrating lymphocyte; TIL, tumour-infiltrating lymphocyte.

Therefore, we further assessed the association between the Rad-TIL model and BCG immunotherapy response within the treatment cohort. The clinicopathological characteristics of the patients in the treatment cohort (*n*=33) are described in the online Supplemental Table S5 (Supplemental Digital Content 2, http://links.lww.com/JS9/D245). Of the 33 patients in the treatment cohort, 23 (69.7%) exhibited an objective response, indicating that they did not experience recurrence after BCG immunotherapy; in contrast, 10 (30.3%) did not respond to BCG immunotherapy and experienced recurrence during the follow-up period. When predicting TIL status in the treatment cohort, the AUC was 0.846 (95% CI: 0.708–0.983; Fig. 6A). After patients were categorised into distinct RS_TIL_ groups, our results revealed that among BCG immunotherapy responders, most patients had high-RS_TIL_ (60.9%, *P*=0.007; Fig. 6B). In contrast, among BCG immunotherapy nonresponders, most patients had low-RS_TIL_ (90.0%, *P*=0.007; Fig. 6B). The AUC of the Rad-TIL model for distinguishing BCG immunotherapy response was 0.728 (95% CI: 0.544–0.913).

## Discussion

Bladder cancer is a malignancy characterised by high recurrence rates and poor prognoses, posing significant challenges in patient management^24–26^. TILs represent a critical subset of immune cells involved in the regulatory control of the TIME^27^. The current and other studies have found that TIL levels in bladder cancer tissue hold significant importance in distinguishing clinicopathological features and predicting prognosis^14,28–30^. TILs are traditionally assessed using paraffin-embedded specimens obtained through invasive procedures such as biopsy or surgery. Nevertheless, because of constraints in the sampling of pathology specimens and the inherent subjectivity of pathologists, TIL evaluation using tissue specimens lacks reproducibility^31^, leading to challenges in the dynamic assessment of TILs as the disease evolves. To the best of our knowledge, this is the first study to integrate tumoral and peritumoral radiomic features and create a noninvasive Rad-TIL model for predicting the TIL status and assessing its predictive value for clinical outcomes, as well as BCG immunotherapy response, in patients with bladder cancer. Moreover, our radiogenomics analysis revealed the potential biological pathways and immune landscape underlying the Rad-TIL model.

TILs, including T, B, and natural killer cells, play a crucial role in bladder cancer TIME and have significant implications for prognosis prediction^28–30,32^. We previously demonstrated that TILs are prognostic indicators of unfavourable survival in patients with bladder cancer^14^. The increased TIL levels in patients with bladder cancer displayed an exhausted phenotype due to the upregulation of the expression of various immune checkpoint molecules, such as PD-L1, programmed death 1 (PD-1), T-cell immunoglobulin and mucin domain-containing protein 3 (TIM-3), and IDO-1^14,23^. These results indicated the need for immunotherapy to reverse the functional impairments of T-cell subpopulations within the TIME in patients with bladder cancer. Notably, we discovered that in our study, a high-TIL status was correlated with a worse prognosis but indicated improved immunotherapy efficacy.

Radiomics is an emerging field involving high-throughput extraction and profiling of quantitative features derived from images^15^. Several studies have demonstrated the potential of radiomics models in predicting bladder cancer prognosis and clinical outcomes^33–35^. We previously demonstrated that the radiomics model is a reliable tool for the assessment of muscular invasiveness and lymph node metastasis in patients with bladder cancer^36,37^. An increasing body of research is emphasising the crucial role of the radiomics features related to the TIME in predicting cancer progression and treatment response in various cancer types, such as gastric and lung cancers^38–40^. Nevertheless, studies assessing the role of radiomics in predicting the TIME in bladder cancer are lacking. The capability of radiomics to capture intricate spatial and textural information, integrate diverse data sources, and reveal concealed biomarkers makes it a superior approach. In the current study, we constructed a novel model based on CT imaging, the Rad-TIL model, to noninvasively predict the TIL status of patients with bladder cancer. The Rad-TIL model could predict TIL status with good performance, with AUCs of 0.852, 0.844, and 0.816 in the training, internal validation, and external validation cohorts, respectively. Furthermore, transcriptome sequencing and IHC analysis confirmed the association between RS_TIL_ and a hot tumour microenvironment.

Most radiomics studies have focused on the analysis of intratumoral features within tumour boundary regions. However, peritumoral features can yield valuable insights into the TIME, including the presence of immune cell infiltration, angiogenesis, and stromal remodelling^40,41^. In reality, integrating peritumoral features into TIME component prediction can significantly enhance the predictive efficacy of radiomic models. Huang *et al*.^40^ reported that a CT prediction model incorporating both tumoral and peritumoral features effectively predicted the neutrophil-to-lymphocyte ratio within the gastric cancer TIME (AUCs range from 0.795 to 0.861). Furthermore, the authors demonstrated a correlation of their CT prediction model with prognosis and anti-PD-1 immunotherapy response^40^. However, there has been a paucity of studies investigating peritumoral features related to TILs in patients with bladder cancer, and relevant conclusive evidence is lacking. In the current study, we developed a radiomics model that integrated both tumoral and peritumoral features in patients with bladder cancer. Notably, in our study, peritumoral features had prospective predictive value for TIL status. The integration of three peritumoral features into Rad-TIL model development resulted in a major improvement in the model’s predictive value.

BCG, a weakened version of *Mycobacterium bovis*, has emerged as a dependable choice in the management of bladder cancer^6^. BCG induces an immune cascade response characterised by immune cell accumulation^42^. The accumulated immune cells release cytokines and chemokines, promoting TIL recruitment^42,43^. In patients with bladder cancer, urinary interleukins 8 and 18 can predict BCG response^43,44^. We previously confirmed the predictive utility of serum CCL27 for BCG immunotherapy response^45^. However, the predictive potential of radiomics for BCG immunotherapy response remains unclear. In the current study, we, for the first time, reported that a high-RS_TIL_ is associated with a high BCG immunotherapy response rate in patients with bladder cancer. Our results may facilitate improved personalised decision-making related to BCG immunotherapy in patients with bladder cancer.

Several studies have evaluated radiomics models’ TIL predictive abilities in various cancer types, such as breast cancer, pancreatic ductal adenocarcinoma, and lung cancer^46–49^. However, previous studies have typically encountered limitations such as a small sample size, a single-centre design, and a lack of comprehensive exploration regarding the association between predicted TIL status and clinical outcomes. In the current study, we enrolled a large number of participants from different centres; thus, our results have the potential to mitigate bias and yield robust, dependable findings.

This study also has a few limitations. First, the present retrospective analyses were performed based on the data from two tertiary hospitals in China. Although quality control and assurance measures were implemented, a degree of selection bias persists, potentially affecting the generalisability of our results. Further validation by researchers from other groups is necessary to confirm our findings. Second, the limited sample size of the BCG immunotherapy cohort may compromise the robustness of our findings. To address this limitation, we are registering a prospective cohort study to validate the results. Finally, we were unable to delve deeper into the mechanisms underlying the interactions between the specific radiomic features and the particular biological processes.

## Conclusion

The Rad-TIL model can accurately and noninvasively predict TIL status in patients with bladder cancer. The predicted TIL status demonstrated a correlation with prognosis and BCG immunotherapy responses. We also identified potential biological pathways and the immune landscape within the Rad-TIL model. These findings may be a valuable addition to TIL status stratification and therapy evaluation systems.

## Ethical approval

This study was approved by the institutional ethical committees of both SYMH (approval number: SYSKY-2023-467-01) and SYUTH (approval number: II2023-303-02).

## Consent

The requirement for informed consent was waived due to this study’s retrospective and anonymous nature.

## Source of funding

This study was supported by the National Key Research and Development Programme of China (Grant No. 2018YFA0902803); the National Natural Science Foundation of China (Grant No. 82000198, 81825016, 81961128027, 81772719, 81772728, 82072831, and 81902582); The Key Areas Research and Development Programme of Guangdong (Grant No. 2018B010109006); the Science and Technology Planning Project of Guangdong Province (Grant No. 2017B020227007; 2022A151501110); Guangdong Provincial Clinical Research Center for Urological Diseases (2020B1111170006); and Medical Scientific Research of Guangdong Province (Grant No. A2019470).

## Author contribution

W.Z. and T.L.: developed the hypothesis and designed study; K.C. and X.L.: designed the study, analysed the data, and wrote the manuscript; L.L. and B.W.: analysed the data and wrote the manuscript; W.L.: revised the manuscript; J.C., M.G., X.H., B.L., and X.S.: performed the immunohistochemical staining and analysed the data; T.Y., X.Z., and W.H.: collected the samples and clinical data; J.H. and Y.L.: modified and revised the manuscript; Y.L., J.H., T.L., and W.Z.: supervised in the design of the study and finalised the manuscript.

## Conflicts of interest disclosure

All authors declare no potential conflicts of interest.

## Research registration unique identifying number (UIN)

1. Name of the registry: ClinicalTrials network.
2. Unique identifying number or registration ID: NCT06381895.
3. Hyperlink to your specific registration (must be publicly accessible and will be checked): https://clinicaltrials.gov/study/NCT06381895?term=NCT06381895&rank=1.

## Guarantor

Wenlong Zhong.

## Data availability statement

The data that support the results of this study can be offered upon request. Correspondence and requests for materials should be addressed to Dr Wenlong Zhong.

## Provenance and peer review

Not involved.

## Supplementary Material
