# Supplementary material for: Correlation of noninvasive imaging of tumour-infiltrating lymphocytes with survival and BCG immunotherapy response in patients with bladder cancer: a multicentre cohort study
Source: Int J Surg. 2024 Aug 5;111(1):920–31. doi: 10.1097/JS9.0000000000001999 (PMC11745626; doi:10.1097/JS9.0000000000001999)
Supplement: Supplementary file 2 [file js9-111-0920-s002.docx]

**Online Supplemental Content**

**Figure S1.** Representative images of stromal TIL in bladder cancer tissue

**Figure S2.** Identification of biological pathways and immune landscape stratified by different TIL groups based on H&E stain

**Figure S3.** Recurrence-free survival outcomes in patients of BCG immunotherapy group and infusion chemotherapy group

**Table S1.** Baseline clinicopathological characteristics of training, internal and external cohorts

**Table S2.** TIL-related features selected to construct the six different TIL predicting models

**Table S3.** Association of RS_TIL_ level with the clinical features of bladder cancer patients in training, internal and external cohorts

**Table S4.** Baseline clinicopathological characteristics of BCG treatment cohort

**Table S5.** Baseline clinicopathological characteristics of radiogenomic cohort from TCIA dataset

**Table S6.** Baseline covariates in patients before and after matching

| **Figure S1. Representative images of stromal TIL in bladder cancer tissue** |
| --- |
| 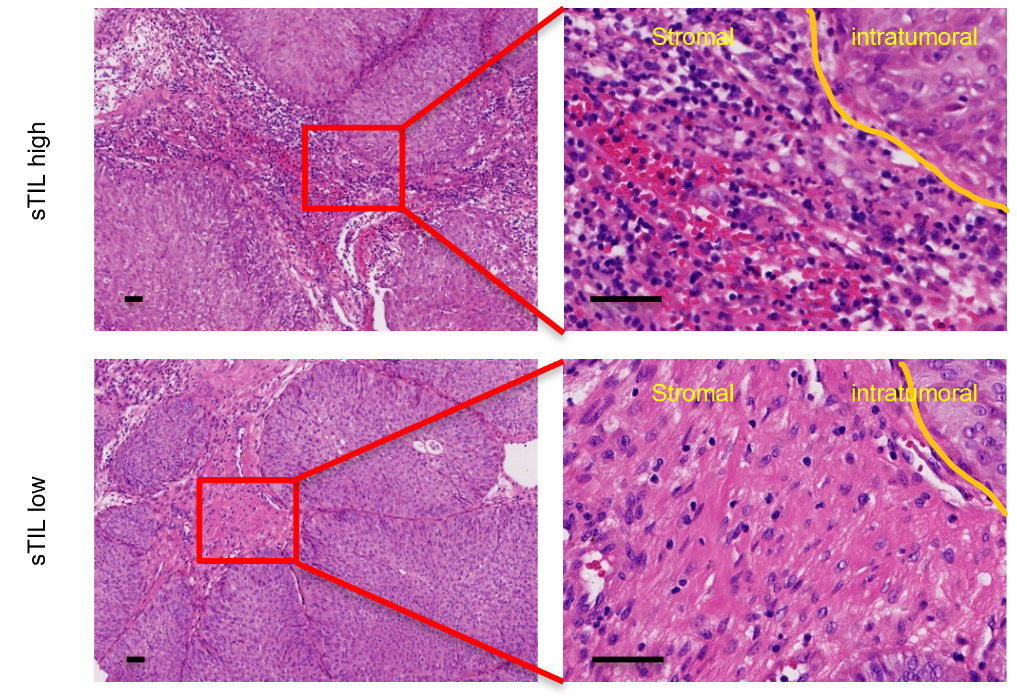 |
| Cases with high TIL in the upper row and cases with low TIL in the lower row. Scale bars indicate 50 μm. Abbreviations: TIL, tumor-infiltrating lymphocyte. |

| **Figure S2. Identification of biological pathways and immune landscape stratified by different TIL groups based on H&E stain** |
| --- |
| 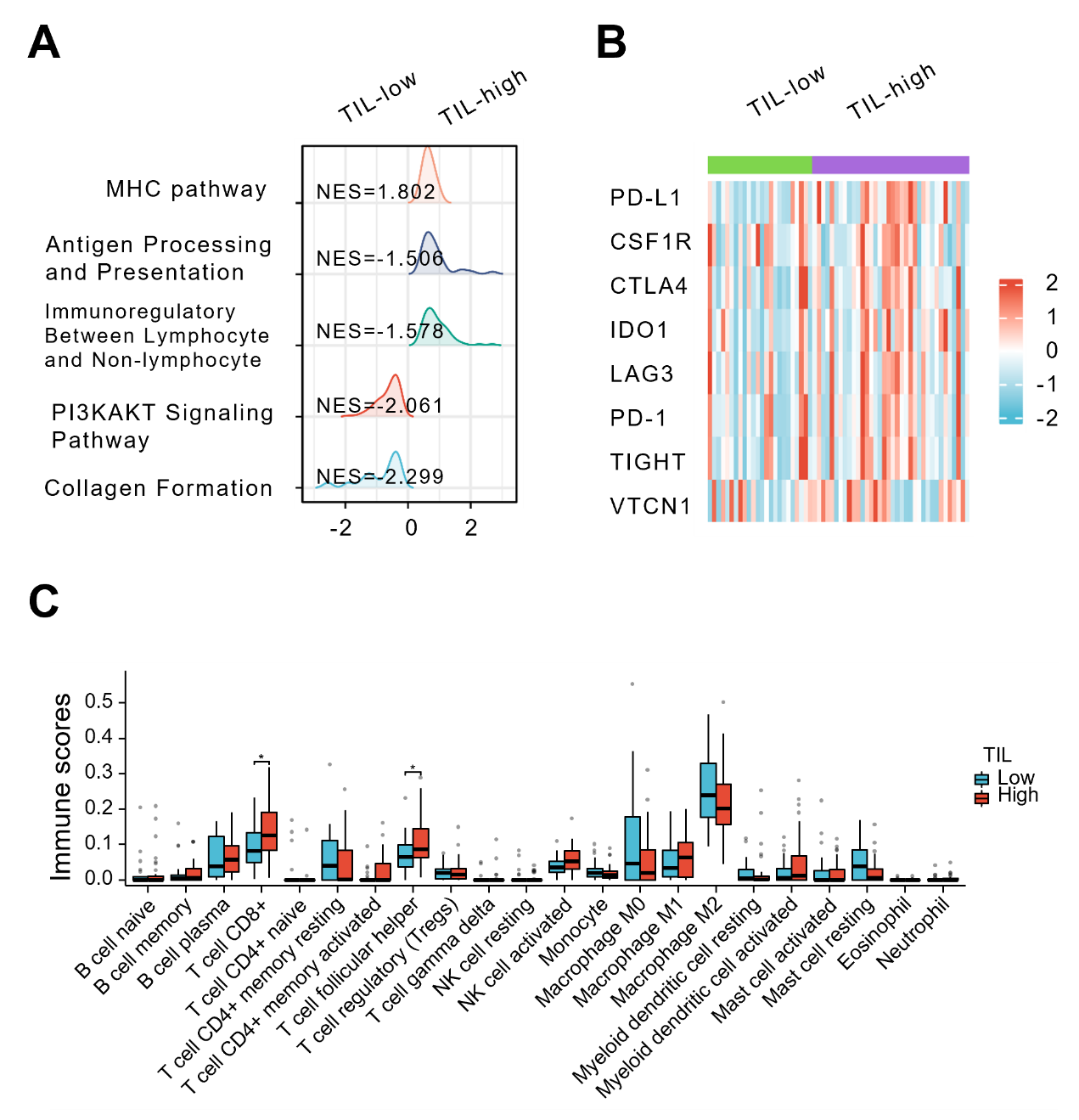 |
| (A) Ridgeline plots of GSEA results showing representative immune-related pathways enrich in different TIL groups in the radiogenomic cohort. (B) Heatmap showing immune checkpoint molecules expression with different TIL groups in the radiogenomic cohort. (C) Analysis of immune cell infiltration differences among different TIL groups using “CIBERSORT” algorithms in the radiogenomic cohort. Abbreviations: TIL, tumor-infiltrating lymphocyte; H&E, hematoxylin-eosin; GSEA, gene set enrichment analysis; |

| **Figure S3. Recurrence-free survival outcomes in patients of BCG** **immunotherapy group and infusion chemotherapy group** |
| --- |
| 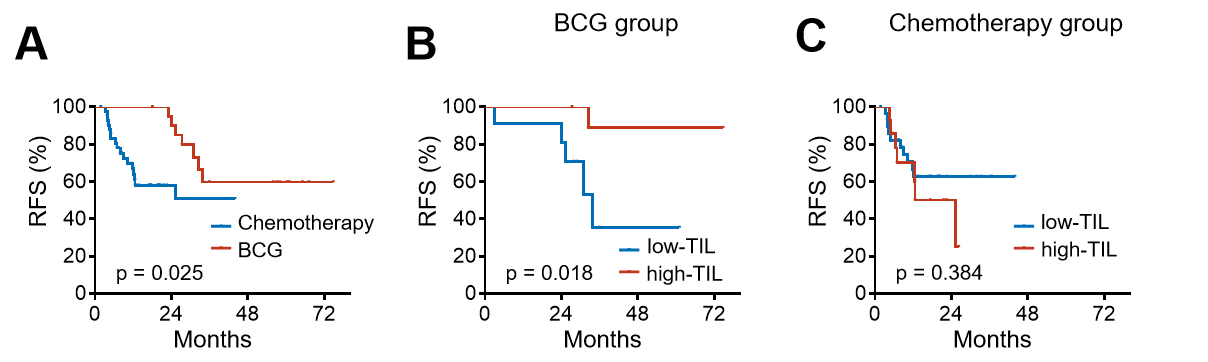 |
| (A) Kaplan–Meier curves depicting RFS in different treatment group. (B, C) Kaplan–Meier curves depicting RFS in different TIL status of BCG immunotherapy group (B) and infusion chemotherapy group (C). Abbreviations: BCG: Bacillus Calmette-Guérin; TIL, tumour-infiltrating lymphocyte; RFS, recurrence-free survival. |

**Table S1. Baseline clinicopathological characteristics of training, internal and external** **cohorts**

| **Variable** | **Training cohort** | **Internal validation cohort** | **External validation cohort** | **p value** |
| --- | --- | --- | --- | --- |
| No. of patients | 258 | 111 | 241 |  |
| Age, years | 62.9±11.8 | 63.0±12.9 | 64.5±12.9 | 0.123 |
| Gender |  |  |  | 0.386 |
| male | 216 (83.7%) | 98 (88.3%) | 199 (82.6%) |  |
| female | 42 (16.3%) | 13 (11.7%) | 42 (17.4%) |  |
| Pathologic T stage |  |  |  | 0.306 |
| Ta-T1 | 185 (71.7%) | 87 (78.4%) | 184 (76.3%) |  |
| T2-T4 | 73 (28.3%) | 24 (21.6%) | 57 (23.7%) |  |
| Pathologic N stage |  |  |  | 0.981 |
| N0 | 245 (95.0%) | 105 (94.6%) | 228 (94.6%) |  |
| N1-N3 | 13 (5.0%) | 6 (5.4%) | 13 (5.4%) |  |
| Histological grade |  |  |  |  |
| Low | 85 (32.9%) | 37 (33.3%) | 96 (39.8%) | 0.233 |
| High | 173 (67.1%) | 74 (66.7%) | 145 (60.2%) |  |
| Tumor size, cm |  |  |  | 0.647 |
| ≤ 3 | 154 (59.7%) | 71 (64.0%) | 152 (63.1%) |  |
| > 3 | 104 (40.3%) | 40 (36.0%) | 89 (36.9%) |  |
| Tumor multifocality |  |  |  | **<0.001** |
| Unifocal | 128 (49.6%) | 53 (47.7%) | 187 (77.6%) |  |
| Multifocal | 130 (50.4%) | 58 (52.3%) | 54 (22.4%) |  |
| Surgical methods |  |  |  | 0.620 |
| TURBT | 179 (69.4%) | 79 (71.2%) | 160 (66.4%) |  |
| Radical cystectomy | 79 (30.6%) | 32 (28.8%) | 81 (33.6%) |  |

Abbreviations: TURBT, transurethral resection of bladder tumor. Significant p-values are shown in **bold**.

**Table S2. TIL-related features selected to construct the six different TIL predicting models**

| **TIL predicting model** | **TIL-related features** | **Coefficient** | |
| --- | --- | --- | --- |
| A_TR model | TR_arterial phase_wavelet-LL_glszm_LargeAreaHighGrayLevelEmphasis  TR_arterial phase_wavelet-HH_gldm_LargeDependenceLowGrayLevelEmphasis  TR_arterial phase_log-sigma-4-0-mm-3D_glcm_Imc1  TR_arterial phase_log-sigma-4-0-mm-3D_glszm_LargeAreaEmphasis  TR_arterial phase_original_glrlm_RunEntropy  TR_arterial phase_original_shape2D_Sphericity  TR_arterial phase_wavelet-LH_glcm_JointEntropy  TR_arterial phase_log-sigma-4-0-mm-3D_glszm_GrayLevelNonUniformity  TR_arterial phase_log-sigma-4-0-mm-3D_glrlm_ShortRunHighGrayLevelEmphasis  TR_arterial phase_wavelet-LH_glszm_SmallAreaHighGrayLevelEmphasis  TR_arterial phase_wavelet-HL_firstorder_Mean  TR_arterial phase_log-sigma-4-0-mm-3D_glcm_ClusterProminence | \| 0.164346 \| \| --- \| \| -0.06248 \| \| 0.19689 \| \| -0.13453 \| \| 0.061135 \| \| -0.13852 \| \| 0.160813 \| \| 0.285776 \| \| 0.29127 \| \| -0.07573 \| \| 0.04199 \| \| -0.03908 \| | |
| A_PR model | \| PR_arterial phase_wavelet-HL_glcm_Correlation \| \| --- \| \| PR_arterial phase_wavelet-LH_glcm_ClusterProminence \| \| PR_arterial phase_log-sigma-4-0-mm-3D_glcm_Imc1 \| | \| 0.018449 \| \| --- \| \| -0.05992 \| \| 0.376349 \| | |
| V_TR model | \| TR_venous phase_log-sigma-4-0-mm-3D_glcm_SumEntropy \| \| --- \| \| TR_venous phase_log-sigma-4-0-mm-3D_glszm_SizeZoneNonUniformity \| \| TR_venous phase_log-sigma-4-0-mm-3D_glszm_LargeAreaHighGrayLevelEmphasis \| \| TR_venous phase_log-sigma-4-0-mm-3D_glcm_Autocorrelation \| \| TR_venous phase_original_glszm_LargeAreaLowGrayLevelEmphasis \| | \| 0.188899 \| \| --- \| \| 0.124254 \| \| -0.05646 \| \| 0.191407 \| \| -0.06094 \| | |
| **TIL predicting model** | **TIL-related features** | **Coefficient** | |
| V_TR model | \| TR_venous phase_wavelet-LH_glcm_InverseVariance \| \| --- \| \| TR_venous phase_wavelet-LH_glcm_DifferenceVariance \| \| TR_venous phase_log-sigma-4-0-mm-3D_glrlm_ShortRunHighGrayLevelEmphasis \| | \| 0.13887 \| \| --- \| \| -0.2434 \| \| 0.080075 \| | |
| V_PR model | \| PR_venous phase_wavelet-LL_ngtdm_Complexity \| \| --- \| \| PR_venous phase_log-sigma-4-0-mm-3D_glszm_LowGrayLevelZoneEmphasis \| \| PR_venous phase_wavelet-LH_glcm_ClusterTendency \| \| PR_venous phase_log-sigma-4-0-mm-3D_glcm_MaximumProbability \| \| PR_venous phase_original_shape2D_Sphericity \| \| PR_venous phase_wavelet-LL_glcm_InverseVariance \| \| PR_venous phase_log-sigma-4-0-mm-3D_glcm_Idmn \| \| PR_venous phase_wavelet-HH_gldm_DependenceEntropy \| \| PR_venous phase_wavelet-LL_glszm_SizeZoneNonUniformityNormalized \| | \| -0.07064 \| \| --- \| \| -0.0953 \| \| -0.17846 \| \| -0.06074 \| \| -0.21765 \| \| 0.04703 \| \| 0.02531 \| \| 0.023616 \| \| -0.06735 \| | |
| A_TR+PR model | \| TR_arterial phase_wavelet-LL_glszm_LargeAreaHighGrayLevelEmphasis \| \| --- \| \| TR_arterial phase_wavelet-HH_gldm_LargeDependenceLowGrayLevelEmphasis \| \| TR_arterial phase_log-sigma-4-0-mm-3D_glszm_LowGrayLevelZoneEmphasis \| \| TR_arterial phase_log-sigma-4-0-mm-3D_glcm_Imc1 \| \| PR_arterial phase_log-sigma-4-0-mm-3D_glszm_GrayLevelNonUniformity \| \| PR_arterial phase_wavelet-HL_glcm_Idm \| \| PR_arterial phase_wavelet-HH_firstorder_InterquartileRange \| \| TR_arterial phase_wavelet-LH_glcm_JointEntropy \| \| TR_arterial phase_log-sigma-4-0-mm-3D_glszm_LargeAreaEmphasis \| \| TR_arterial phase_original_glrlm_RunEntropy \| | \| 0.117256 \| \| --- \| \| -0.09947 \| \| -0.2796 \| \| 0.168885 \| \| 0.016513 \| \| 0.095921 \| \| -0.0792 \| \| 0.012432 \| \| -0.23571 \| \| 0.045626 \| | |
| **TIL predicting model** | **TIL-related features** | | **Coefficient** |
| A_TR+PR model | \| PR_arterial phase_wavelet-LL_gldm_SmallDependenceHighGrayLevelEmphasis \| \| --- \| \| TR_arterial phase_log-sigma-4-0-mm-3D_glcm_ClusterShade \| \| TR_arterial phase_log-sigma-4-0-mm-3D_glszm_SizeZoneNonUniformity \| \| PR_arterial phase_wavelet-HL_glcm_Correlation \| \| PR_arterial phase_wavelet-LH_glcm_ClusterProminence \| | | \| -0.00508 \| \| --- \| \| -0.15076 \| \| 0.077026 \| \| 0.110407 \| \| -0.16008 \| |
| V_TR+PR model | \| TR_venous phase_log-sigma-4-0-mm-3D_glcm_SumEntropy \| \| --- \| \| PR_venous phase_log-sigma-4-0-mm-3D_glszm_LowGrayLevelZoneEmphasis \| \| PR_venous phase_wavelet-LL_ngtdm_Complexity \| \| TR_venous phase_log-sigma-4-0-mm-3D_glcm_Autocorrelation \| \| TR_venous phase_wavelet-LH_gldm_GrayLevelVariance \| \| TR_venous phase_original_glszm_LargeAreaLowGrayLevelEmphasis \| \| TR_venous phase_wavelet-LH_glcm_InverseVariance \| \| TR_venous phase_log-sigma-4-0-mm-3D_glszm_SizeZoneNonUniformity \| \| TR_venous phase_log-sigma-4-0-mm-3D_glszm_LargeAreaHighGrayLevelEmphasis \| \| PR_venous phase_wavelet-LH_glcm_ClusterTendency \| | | \| 0.20091 \| \| --- \| \| -0.04943 \| \| -0.04266 \| \| 0.249226 \| \| -0.13856 \| \| -0.05234 \| \| 0.122188 \| \| 0.07778 \| \| -0.07104 \| \| -0.17007 \| |

Abbreviations: TIL, tumor-infiltrating lymphocyte; A: arterial phases; V: venous phases; TR: tumoral region; PR: peritumoral region

**Table S3. Association of RS_TIL_ level with the clinical features of bladder cancer patients in training, internal and external cohorts**

|  | **Training cohort** | | |  | **Internal validation cohort** | | |  | **External validation cohort** | | |
| --- | --- | --- | --- | --- | --- | --- | --- | --- | --- | --- | --- |
| **Variable** | **Low** | **High** | **p** |  | **Low** | **High** | **p** |  | **Low** | **High** | **p** |
| No. of patients | 161 | 97 |  |  | 60 | 51 |  |  | 188 | 53 |  |
| Age, years |  |  | 0.520 |  |  |  | 0.974 |  |  |  | 0.605 |
| Mean, SD | 63.0 ± 12.0 | 64.0 ± 11.5 |  |  | 62.8 ± 12.3 | 62.9 ± 10.1 |  |  | 64.7 ± 12.6 | 63.7 ± 13.7 |  |
| Gender |  |  | 0.527 |  |  |  | 0.353 |  |  |  | 0.082 |
| Male | 136 (52.7%) | 79 (30.6%) |  |  | 52 (46.8%) | 47 (42.3%) |  |  | 151 (62.7%) | 48 (19.9%) |  |
| Female | 25 (9.7%) | 18 (7%) |  |  | 8 (7.2%) | 4 (3.6%) |  |  | 37 (15.4%) | 5 (2.1%) |  |
| Tumor size, cm |  |  | **< 0.001** |  |  |  | 0.002 |  |  |  | **< 0.001** |
| ≤ 3 | 119 (46.1%) | 37 (14.3%) |  |  | 45 (40.5%) | 24 (21.6%) |  |  | 141 (58.5%) | 11 (4.6%) |  |
| > 3 | 42 (16.3%) | 60 (23.3%) |  |  | 15 (13.5%) | 27 (24.3%) |  |  | 47 (19.5%) | 42 (17.4%) |  |
| Multifocality |  |  | 0.999 |  |  |  | 0.918 |  |  |  | 0.534 |
| Unifocal | 78 (30.2%) | 47 (18.2%) |  |  | 30 (27%) | 26 (23.4%) |  |  | 145 (60.2%) | 43 (17.8%) |  |
| Multifocal | 83 (32.2%) | 50 (19.4%) |  |  | 30 (27%) | 25 (22.5%) |  |  | 43 (17.8%) | 10 (4.1%) |  |
| Pathologic T stage |  |  | **< 0.001** |  |  |  | 0.071 |  |  |  | **< 0.001** |
| Ta-T1 | 143 (55.4%) | 48 (18.6%) |  |  | 48 (43.2%) | 33 (29.7%) |  |  | 154 (63.9%) | 30 (12.4%) |  |
| T2-T4 | 18 (7%) | 49 (19%) |  |  | 12 (10.8%) | 18 (16.2%) |  |  | 34 (14.1%) | 23 (9.5%) |  |
| Pathologic N stage |  |  | **< 0.001** |  |  |  | 0.886 |  |  |  | **0.041** |
| N0 | 158 (61.2%) | 84 (32.6%) |  |  | 59 (53.2%) | 49 (44.1%) |  |  | 182 (75.5%) | 47 (19.5%) |  |
| N1-N3 | 3 (1.2%) | 13 (5%) |  |  | 1 (0.9%) | 2 (1.8%) |  |  | 6 (2.5%) | 6 (2.5%) |  |
| Histological grade |  |  | **< 0.001** |  |  |  | 0.973 |  |  |  | **< 0.001** |
| Low | 70 (27.1%) | 17 (6.6%) |  |  | 19 (17.1%) | 16 (14.4%) |  |  | 88 (36.5%) | 9 (3.7%) |  |
| High | 91 (35.3%) | 80 (31%) |  |  | 41 (36.9%) | 35 (31.5%) |  |  | 100 (41.5%) | 44 (18.3%) |  |

Abbreviations: RS_TIL_: radiomics score of tumor-infiltrating lymphocytes; SD: standard deviation. Significant p-values are shown in **bold**.

**Table S4. Baseline clinicopathological characteristics** **of BCG treatment cohort**

| **Variable** | **BCG treatment cohort** |
| --- | --- |
| No. of patients | 33 |
| Age, years |  |
| Mean, SD | 60.9 ± 13.8 |
| Gender |  |
| male | 29 (87.9%) |
| female | 4 (12.1%) |
| Pathologic T stage |  |
| Ta | 22 (66.7%) |
| T1 | 11 (33.3%) |
| Histological grade |  |
| Low | 12 (36.4%) |
| High | 21 (63.6%) |
| Tumor size, cm |  |
| ≤ 3 | 28 (84.8%) |
| > 3 | 5 (15.2%) |
| Tumor multifocality |  |
| Unifocal | 21 (63.6%) |
| Multifocal | 12 (36.4%) |
| Response status |  |
| Failure | 8 (24.2%) |
| Response | 25 (75.8%) |

Abbreviations: BCG: Bacillus Calmette-Guérin; SD: standard deviation.

**Table S5. Baseline clinicopathological characteristics of radiogenomic cohort from TCIA dataset**

| **Variable** | **Radiogenomic cohort** |
| --- | --- |
| No. of patients | 60 |
| Age, years |  |
| Mean, SD | 70.2 ± 10.1 |
| Gender |  |
| male | 50 (83.3%) |
| female | 10 (16.7%) |
| Pathologic T stage |  |
| Ta-T1 | 0 (0%) |
| T2-T4 | 59 (98.3%) |
| NA | 1 (0.7%) |
| Pathologic N stage |  |
| N0 | 33 (55.0%) |
| N1-N3 | 15 (25.0%) |
| NA | 12 (20.0%) |
| Histological grade |  |
| Low | 0 (0%) |
| High | 60 (100%) |

Abbreviations: TCIA: The Cancer Imaging Archive; SD: standard deviation.

**Table S6. Baseline covariates in patients before and after matching**

| **Variables** | **Level** | **Before Matching** | | |  | **After Matching** | | |
| --- | --- | --- | --- | --- | --- | --- | --- | --- |
|  |  | **Chemotherapy** | **BCG treatment** | **SMD^△^** |  | **Chemotherapy** | **BCG treatment** | **SMD^△^** |
| n |  | 296 | 21 |  |  | 42 | 21 |  |
| Age, years |  | 65.46 (11.21) | 61.05 (16.37) | -0.270 |  | 61.31 (13.05) | 61.05 (16.37) | -0.016 |
| Tumor size, cm |  | 32.32 (15.93) | 29.29 (10.88) | -0.279 |  | 30.02 (15.62) | 29.29 (10.88) | -0.067 |
| Multifocality | Unifocal | 175 (59.1) | 17 (81.0) | 0.556 |  | 33 (78.6) | 17 (81.0) | 0.061 |
|  | Multifocal | 121 (40.9) | 4 (19.0) | -0.556 |  | 9 (21.4) | 4 (19.0) | -0.061 |
| Gender | Male | 253 (85.5) | 20 (95.2) | 0.459 |  | 39 (92.9) | 20 (95.2) | 0.112 |
|  | Female | 43 (14.5) | 1 (4.8) | -0.459 |  | 3 (7.1) | 1 (4.8) | -0.112 |
| Pathologic T stage | Ta | 34 (11.5) | 11 (52.4) | 0.819 |  | 21 (50.0) | 11 (52.4) | 0.048 |
|  | T1 | 138 (46.6) | 10 (47.6) | 0.020 |  | 21 (50.0) | 10 (47.6) | -0.048 |
|  | T2 | 74 (25.0) | 0 (0.0) | -0.597 |  | 0 (0.0) | 0 (0.0) | 0.000 |
|  | T3 | 33 (11.1) | 0 (0.0) | -0.367 |  | 0 (0.0) | 0 (0.0) | 0.000 |
|  | T4 | 17 (5.7) | 0 (0.0) | -0.255 |  | 0 (0.0) | 0 (0.0) | 0.000 |
| ^△^Standardized Mean Difference | | | | | | | | |
